# Lactic acid bacteria in the meat industry: flavor, function, and food safety

**DOI:** 10.3389/fmicb.2025.1703213

**Published:** 2025-11-13

**Authors:** Bhagavathi Sundaram Sivamaruthi, Periyanaina Kesika, Safreena Barwin Syed Rafeek, Kasthuri Sivakumar, Shanmuga Priya Ramasamy, Chaiyavat Chaiaysut, Pranom Fukngoen, Karthikeyan Alagarsamy

**Affiliations:** 1 Faculty of Pharmacy, Innovation Center for Holistic Health, Nutraceuticals, and Cosmeceuticals, Chiang Mai University, Chiang Mai, Thailand; 2 Office of Research Administration, Chiang Mai University, Chiang Mai, Thailand; 3 Department of Microbiology, Karpagam Academy of Higher Education, Coimbatore, India; 4 Department of Microbiology, PSG College of Arts and Science, Coimbatore, Tamil Nadu, India

**Keywords:** meat products, lactic acid bacteria, food safety, bacteriocins, preservation

## Abstract

Lactic acid bacteria (LAB) play a pivotal role in the food industry, particularly in the fermentation and preservation of meat products. These Gram-positive, non-spore-forming microorganisms contribute significantly to food safety, shelf-life extension, and sensory quality enhancement through the production of various bioactive compounds, including organic acids, bacteriocins, exopolysaccharides, and gamma-aminobutyric acid. Their antimicrobial and probiotic properties are attributed to inhibiting the growth of spoilage organisms and foodborne pathogens, thereby reducing the reliance on synthetic preservatives. This review discusses the general characteristics and selection criteria of LAB, with a focus on their biochemical contributions to the development of flavor, texture, and functional properties in meat-based products. LABs are increasingly being recognized for their potential as natural bio-preservatives, aligning with the growing consumer demand for clean-label and functional foods. However, several challenges persist, including strain-specific variability in functional properties, safety assessments, optimization of metabolite production, and consumer perception. Addressing these limitations through multidisciplinary research and technological innovation is essential to enhance the effective and sustainable application of LAB in the meat industry.

## Graphical abstract

**Figure:**
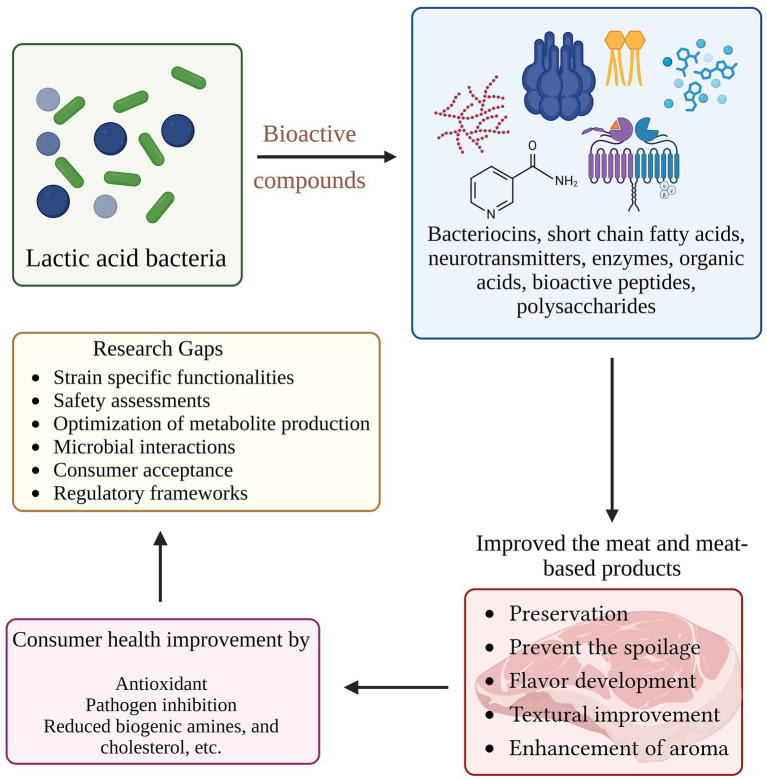
Bioactive compounds produced by lactic acid bacteria and their roles in meat and meat-based products, highlighting consumer health benefits and current research gaps.

## 1 Introduction

Lactic acid bacteria (LAB) have long been recognized for their significant role in the food industry, particularly in fermentation, preservation, and quality enhancement. They are non-spore-forming bacteria belonging to genera including *Lactiplantibacillus, Leuconostoc,* and *Streptococcus.* They are classified as obligatory or relative anaerobes, which gives them the ability to withstand the acidic environmental conditions (Gupta et al., 2018). These microorganisms are primarily known for their ability to convert carbohydrates into lactic acid; this metabolic process allows them to produce organic compounds other than lactic acid, such as mannitol and dextran. These organic compounds play a crucial role in extending shelf life, enhancing safety, and improving the sensory properties of various food products (Mesele, 2018). To produce fermented dairy products, such as yogurt, cheese, butter, and sour cream, lactic fermentation is utilized to acidify milk. Furthermore, this method is utilized for cold cut maturation, and it is responsible for producing and stabilizing sourdough and vegetable silage (Muhialdin et al., 2020). As these LAB are widely used in the food industry, they are usually regarded as safe; additionally, they are an essential part of the natural microflora found in the human intestine (Agriopoulou et al., 2020). Among their many applications, their role in the meat industry is particularly significant. Meat and meat products are rich in nutrients like protein, fats, vitamins, and minerals; they have long been a staple of human diets. Meat, however, also serves as a significant source of nutrients for pathogenic bacteria, which can proliferate quickly, leading to increased food waste and financial losses for the meat industry (Woraprayote et al., 2016). Microbial contamination is therefore a significant concern regarding quality and safety in the meat industry (Pradhan et al., 2018). Thus, the meat industry employs a range of traditional techniques, including drying, freezing, packaging, canning, curing, and dehydration, as well as chemical treatment processes, to create safe food items with extended shelf life. Over the past several years, there has been a growing demand for high-quality meats and food products with high nutritional content, free from synthetic chemicals (Zhaxybayeva et al., 2020).

To address these issues, the food manufacturing sector is seeking innovative natural alternatives that serve as preservatives, ensure sufficient microbiological safety, and prolong the shelf life of products. According to numerous studies, a small number of microorganisms from the LAB can be added or utilized as bio-protective cultures or starters in meat-based products (Bintsis, 2018). LAB can stop the growth and other reactions of spoilage bacteria, possibly due to their metabolites, antimicrobial compounds, which help prevent meat degradation. LAB also has prospects as efficient and natural food preservatives, and a suitable alternative to chemicals. Additionally, LAB strains have been explored for their probiotic potential and ability to produce bioactive compounds, further increasing their value in functional meat products (Imade et al., 2021).

## 2 General characteristics and selection process of lactic acid bacteria

LAB are microaerophilic organisms and prefer anaerobic environments for their growth. Most of the LAB strains prefer an acidic pH (Strafella et al., 2020). More than 25 genera, including *Schleiferilactobacillus, Lacticaseibacillus, Levilactobacillus, Agrilactobacillus, Furfurilactobacillus, Fructilactobacillus, Lactiplantibacillus, Ligilactibacillus, Paralactobacillus, Streptococcus, Carnobacterium, Enterococcus, Pediococcus,* and *Weissella,* are considered as LAB (Da Costa et al., 2019) (Table 1). The genus *Lactobacillus* was split into 25 genera (e.g., *Lacticaseibacillus*, *Lactiplantibacillus*, *Levilactobacillus*) in 2020 due to its considerable diversity. To avoid confusion, new standard abbreviations have been proposed for scientific use, while also allowing references to the “former *Lactobacillus*” when discussing older studies (Zheng et al., 2020; Todorov et al., 2023). LAB plays an important role in food fermentations. Fermentation occurs with the participation of homo and hetero-fermentative LAB. Homo-fermentation is a mechanism by which certain LAB convert disaccharides into nearly pure lactic acid. Another slightly different process is called hetero-fermentation, wherein lactic acid is not the sole by-product of lactose breakdown, but also produces ethyl alcohol, carbon dioxide, hydrogen peroxide, diacetyl, acetoin, and acetic aldehyde (Mandha et al., 2021). LAB are crucial as they produce a variety of metabolites with antimicrobial activity during the growth and fermentation process, such as lactic acid, acetic acid, hydrogen peroxide, low molecular weight compounds (diacetyl, fatty acids, reuterin, reutericyclin), antifungal substances (phenyl lactate, propionate, hydroxyphenyl lactate), and bacteriocins (Castellano et al., 2017). LABs are primarily found in environments rich in nutrients. They are a significant component of the microbial communities present in dairy products, such as milk, cheeses, and kefir, as well as in fish, meat, and vegetables. They are also a part of the natural microbiota of the gastrointestinal tract and the vagina of both humans and animals (Bengoa et al., 2019). LAB have been utilized as starters, adjuncts, and protective microorganisms in the production of fermented meats, vegetables, dairy products (such as yogurt and cheese), and fish products (Ashaolu, 2020; Ashaolu and Reale, 2020; Peerajan et al., 2016; Woraharn et al., 2016) (Table 2).

**Table 1:** Commonly used lactic acid bacteria in meat preservation (Kaveh et al., 2023).

Genus	Species
*Lactobacillus*	*Lactobacillus delbrueckii*, *Lactobacillus bulgaricus*, *Lactobacillus gallinarum*, *Lactobacillus gasseri, Lactobacillus lactis*, *Lactobacillus helveticus*, *Lactobacillus reuteri*, *Lactobacillus acidophilus, Lactobacillus curvatus*, *Lactobacillus sakei*, *Lactobacillus salivarius*
*Lactiplantibacillus*	*Lactiplantibacillus pentosus*, *Lactiplantibacillus plantarum*, *Lactiplantibacillus brevis*, *Lactiplantibacillus casei*
*Lacticaseibacillus*	*Lacticaseibacillus paracasei*, *Lacticaseibacillus rhamnosus*, *Lacticaseibacillus casei*
*Pediococcus*	*Pediococcus acidilactici*, *Pediococcus pentosaceus*, *Pediococcus parvulus*
*Leuconostoc*	*Leuconostoc mesenteroides*, *Leuconostoc citreum*, *Leuconostoc pseudomesenteroides*, *Leuconostoc carnosum*
*Latilactobacillus*	*Leuconostoc sakei*, *Leuconostoc curvatus*
*Limosilactobacillus*	*Limosilactobacillus fermentum*, *Limosilactobacillus reuteri*

**Table 2:** Lactic acid bacteria isolated from meat and fish products and their possible applications.

Name of the strains		Study details	Food type/samples	Application/Spectrum of action	References
Old name	Updated				
*Lactobacillus sakei* and *Lactobacillus plantarum*	*Latilactobacillus sakei* and *Lactiplantibacillus plantarum*	Strains were isolated from fermented meat and studied for autoinducer-2 and LuxS properties	Chinese fermented meat	Important in the regulation of microbial succession in fermented meat	Lin et al. (2015)
*Weissella hellenica* BCC 7239	Unchanged	Strains were isolated from fermented pork sausage, and bacteriocin production was evaluated *in vitro*	Fermented pork sausage	Inhibits *Pseudomonas aeruginosa, Salmonella enterica* serovar Typhimurium, *Aeromonas hydrophila*, and *Escherichia coli*	Woraprayote et al. (2015)
*Lactobacillus plantarum* and *Lactobacillus curvatus*	*Lactiplantibacillus plantarum* and *Latilactobacillus curvatus*	Strains were isolated from fermented sausages, and bacteriocin production was evaluated *in vitro*	Fermented sausages and salami	Capable of controlling the growth of *Listeria monocytogenes* Aids in the taste and flavor of fermented sausages. The strain has been considered as a starter culture	Casaburi et al. (2016)
*Lactococcus lactis* spp. *Lactis* and *Lactobacillus sakei*	*Lactococcus lactis* spp. *Lactis* and *Latilactobacillus sakei*	*In vitro* evaluation of anti-*Leuconostoc mesenteroides* activity Group 1: 50 g of bacon + 3 log CFU/g of *Leuconostoc mesenteroides;* Group 2: 50 g of bacon + 3 log CFU/g of *Leuconostoc mesenteroides* and *Lactococcus lactis* spp. *Lactis* (1:1 ratio); Group 3: 50 g of bacon + 3 log CFU/g of *Leuconostoc mesenteroides* and *Latilactobacillus sakei* (1:1 ratio). The samples were vacuum-packed and stored for 90 days at 4 ± 2 °C, then studied	Cooked bacon	It inhibits the growth of pathogenic microorganisms, such as *Leuconostoc mesenteroides*	Comi et al. (2016)
*Pediococcus acidilactici* KTU05, *Pediococcus acidilactici* KTU05-9, *Lactobacillus sakei* KTU05-6	*Pediococcus acidilactici* KTU05, *Pediococcus acidilactici* KTU05-9, *Lactiplantibacillus sakei* KTU05-6	Studied the effect of fermented potato tuber juice-based marination in pork; Meat and marinade ratio 1:1; stored in the refrigerator for 24 h	Pork	Prevent meat discoloration and microbial spoilage, thus increasing the acceptability and shelf-life of meat products	Mozuriene et al. (2016)
*Lactobacillus plantarum* GS16, *Lactobacillus paraplantarum* GS54	*Lactiplantibacillus plantarum* GS16, *Lactiplantibacillus paraplantarum* GS54	Strains were isolated from ham and evaluated for growth, bacteriocin production, partial characterization, antibiotic resistance and virulence factors	Ham	Antimicrobial activity against Gram-positive and Gram-negative bacteria	Anacarso et al. (2017)
*Lactobacillus plantarum* M1-UVs300	*Lactiplantibacillus plantarum* M1-UVs300	Purification of bacteriocin-M1-UVs300 and characterization	Chinese fermented sausage	Antimicrobial activity against Gram-positive and Gram-negative bacteria	An et al. (2017)
*Lactobacillus alimentarius* FM-MM4	*Companilactobacillus alimentarius* FM-MM4	The strain produces Lactocin MM4 (molecular mass 1104.58 Da); it has thermostable and broad-spectrum antimicrobial activity	Fermented meat Product (Nanx Wudl)	Antimicrobial activity against Gram-positive and Gram-negative bacteria and Yeasts (*Saccharomyces cerevisiae, Pichia* sp., *Candida albicans*)	Hu et al. (2017)
*Lactobacillus plantarum* DY4-2	*Lactiplantibacillus plantarum* DY4-2	Bacteriocin was purified and characterized. Bacteriocin showed broad-spectrum antimicrobial activity	Fish	Antimicrobial activity against *Pseudomonas fluorescens, Pseudomonas aeruginosa, Vibrio harveyi, Bacillus cereus, Shewanella putrefaciens, Psychrobacter* sp., *Bacillus licheniformis, Listeria monocytogenes*	Lv et al. (2018)
*Lactobacillus reuteri* or *Lactobacillus plantarum*	*Limosilactobacillus reuteri* or *Lactiplantibacillus plantarum*	Studied the antimicrobial activity, chemical and sensory changes in ground beef	Ground beef	Antimicrobial activity against *Listeria monocytogenes*	Khalili Sadaghiani et al. (2019)
*Lactobacillus sakei* ST153	*Latilactobacillus sakei* ST153	Effect of modified atmosphere packaging on anti-*Listeria* activity and sensory attributes	cured smoked pork loin	Antimicrobial activity against *Listeria* spp.	Casquete et al. (2019)
*Lactobacillus curvatus* UFV-NPAC1	*Latilactobacillus curvatus* UFV-NPAC1	UFV-NPAC1 (10^6^ CFU/g) was mixed with pork mixture and stored at 25 °C for 2 h, then sausages were prepared and stored at 7 °C for 10 days. The sausages were studied for their physicochemical properties	Fresh pork sausage	Antimicrobial activity against *Listeria monocytogenes*	De Castilho et al. (2020)
*Lactobacillus paracasei* subsp. tolerans N2 and *Lactobacillus casei* subsp. casei TM1B	*Lacticaseibacillus paracasei* subsp. tolerans N2 and *Lacticaseibacillus casei*	Biosurfactant produced by the LB strains inhibits the microbes. 3 kg of meat soaked in 1 L of bacterial mix containing 7 log CFU/ml of each strain for 1 h	Raw ground goat meat	Reduce the total aerobic microbial counts and are active against *Escherichia coli* MTCC 118 and *Pseudomonas aeruginosa* MTCC 1934. Stabilize the color of goat meat and prevent lipid peroxidation. Potent biopreservatives for goat meat	Mouafo et al. (2020a)
*Lactobacillus sakei* CTC494	*Latilactobacillus sakei* CTC494	Effect of CTC494 on *Listeria monocytogenes* during fermentation and ripening of chicken sausages Minced chicken was mixed with 6 Log_10_ CFU/g of *Listeria* (mixed for 75 Sec) and CTC494 (mixed for 135 Sec), made sausage and assessed the Physicochemical characteristics of both strains	Chicken-based dry-fermented sausages	Protect against *Listeria monocytogenes* during fermentation and ripening	Austrich-Comas et al. (2022)
*Lactobacillus plantarum* 1-24-LJ	*Lactiplantibacillus plantarum* 1-24-LJ	Strain was 1-24-LJ (7 log CFU/g) with or without lipase (50 U/g) in fish batter (Fish, 35% rice flour, and 3% salt) and studied for the physiochemical and microbial diversity at different durations	Chinese fermented fish product (Suanzhayu)	Reduces spoilage bacteria like *Proteobacteria, Escherichia coli, Salmonella,* and enhances product quality and reduces the fermentation time	Zhang et al. (2023)

According to the European Food Safety Authority and the Food and Drug Administration (FDA), LABs are considered Generally Recognized as Safe (GRAS), which means they are safe for consumption by humans and animals. LAB could be obtained from various sources, including decomposing sites, dairy and other fermented food products, animal and human gut, mouth cavities, and agroecosystems (Raman et al., 2022). The commonly used LABs are *Lactiplantibacillus, Lacticaseibacillus, Latilactobacillus, Limosilactobacillus*, *Lactococcus, Leuconostoc, Pediococcus, Weissella*, and *Periweissella* (Ayivi et al., 2020).

The initial screening and selection process of LAB involves several key factors, including immunogenicity, phenotype, and genotype stability (including plasmid stability), carbohydrate and protein utilization patterns, production of antimicrobial substances, and the capacity to inhibit known pathogens, spoilage organisms, or both. LAB can be isolated from various sources; however, to be used for human use, it must be safe and isolated from the human microflora system (Bikila, 2015).

The process of selecting LAB involves a comprehensive evaluation from four main perspectives: safety, technology, functionality, and benefits. The main goal of safety aspects is to identify and describe the bacterial strain’s species, genus, and place of origin. Assessing the strain’s pathogenicity and infectivity, as well as its virulence factors (including metabolic activity, toxicity, and inherent features such as antibiotic resistance), is essential for ensuring consumer safety. Technological aspects examine the strain’s stability and performance during production and storage. Genetic stability, excellent viability throughout processing, and the addition of desirable sensory attributes to the finished product are all characteristics of ideal strains. Functional characteristics assess a strain’s ability to endure difficult gastrointestinal conditions, including exposure to bile acids, low pH, and gastric and pancreatic secretions. Benefits revolve around the strain’s ability to suppress dangerous microorganisms and alter the immune system (Gupta et al., 2018).

## 3 Bioactive compounds from lactic acid bacteria

A range of physical and chemical preservation techniques was employed to inhibit the pathogenic microbial growth and to increase the shelf life of meat products (Kaveh et al., 2023). However, most physical and chemical methods are associated with various drawbacks, including nutritional alterations and changes in the organoleptic properties of meat products. Moreover, the excessive consumption of these chemical preservatives causes carcinogenic effects in humans. Thus, the needs for bio-preservatives in the food industry possess significant importance and consumer interest to produce chemical-free food products (Kaveh et al., 2023; Gómez et al., 2020). Among the key LAB species involved in the processing of meat products are *Latilactobacillus sakei, Latilactobacillus curvatus,* and *Lactiplantibacillus plantarum* (Parlindungan et al., 2021). LAB plays a significant role in meat safety and protection through the production of various bioactive compounds. The capacity of lactic acid bacteria to generate significant quantities of bioactive compounds during fermentation is well established. The most significant bioactive substances produced by LAB during fermentation are peptides, EPS, bacteriocins, vitamins, gamma-aminobutyric acid, some amylases, proteases, lipase enzymes, and lactic acid (Fitsum et al., 2025; Anumudu et al., 2024). The health-promoting qualities of LAB make them useful microorganisms. Thus, LAB ensures the consumption of safe and nutritious food for all human beings (Perez and Ancuelo, 2023).

### 3.1 Bacteriocins

Active metabolic peptides known as bacteriocins are produced by the ribosome of specific LAB and non-lactic acid bacteria. Different LAB produce distinct bacteriocins, each with its own unique biochemical, structural, genetic, ecological, and metabolic properties (Balay et al., 2017; Choeisoongnern et al., 2020). The role of bacteriocins includes causing damage to the integrity of the target bacteria’s cells, impeding biological functions, and interfering with DNA or protein synthesis. Bacteriocin production is significantly influenced by several environmental parameters, including pH, incubation temperature, nutritional availability, and the composition of the growth medium (Kumariya et al., 2019). Generally, bacteriocins are positively charged molecules and are hydrophobic. Bacteriocins can interact with the negatively charged microbial membranes (phosphate groups) or the receptors present in the bacterial cell wall. Bacteriocins can target nucleic acid synthesis and protein synthesis in pathogenic bacteria and affect the balance of the cytoplasmic membrane. Furthermore, bacteriocins produce pores in the cell membranes of pathogenic bacteria, which ultimately affect the pH of the target cell and cause cellular material leakage (Kaveh et al., 2023). Bacteriocins typically display a narrow antimicrobial spectrum, often inhibiting microorganisms that are phylogenetically related (closely related species or genera) to the producing strain (Riley and Wertz, 2002; Cotter et al., 2005). In addition, Sakacin Q is a bacteriocin that was produced by *Latilactobacillus curvatus* ACU-1 (formerly *Lactobacillus curvatus*) isolated from artisanal dry sausages and can inhibit *Listeria monocytogenes* on cooked meat products (Rivas et al., 2014). Bacteriocins are classified into class I, class II, class III, and class IV bacteriocins (Table 3).

**Table 3:** Comparison of bacteriocin classes.

Class	Defining criteria	Exemplars	Antimicrobial activity	Current status
I (lantibiotics)	<5 kDa; Post-translationally modified; Unusual amino acids (lanthionine, methyllanthionine)	Nisin, epidermin, gallidermin	Strong pore-forming; active against Gram+ bacteria	Accepted
II (small non-lantibiotics)	<10 kDa; Heat-stable; Amphiphilic helices; Subclasses IIa–IId	Pediocin PA-1, leucocin A, plantaricin NC8	Potent, esp. anti-*Listeria*	Accepted
III (large non-lantibiotics)	>30 kDa; Heat-labile proteins	Helveticin, enterolysin A	Lytic, cell wall-degrading	Accepted
IV (complex conjugates)	Protein + lipid or carbohydrate moieties	Lipoprotein- or glyco-conjugated peptides (rare)	Inconsistent; often poorly defined	Controversial

Bacteriocins exhibit strong activity against foodborne pathogens, such as *Listeria monocytogenes, Clostridium* spp., and *Staphylococcus aureus*, and have demonstrated stability across a range of pH values, temperatures, and storage conditions, thereby enhancing their technological applicability. Importantly, bacteriocinogenic LAB such as *Latilactobacillus sakei* (formerly *Lactobacillus sakei*) and *Latilactobacillus curvatus* (formerly *Lactobacillus curvatus*) have been widely isolated from fermented meat products and are recognized for producing sakacins and curvacins with potent anti-listerial activity, thus underscoring the potential of meat-derived strains in food biopreservation. Bacteriocins can be applied to meat products by adding them directly, incorporating them into antimicrobial packaging, or using bacteriocin-producing LAB as starter or protective cultures. Evidence suggests that bacteriocins not only inhibit spoilage and pathogenic microorganisms but also contribute positively to product shelf life and sensory quality. Nevertheless, limitations such as reduced efficacy against Gram-negative bacteria, possible inactivation by meat matrix components, and regulatory barriers must be addressed. It has been recognized that bacteriocins should be integrated into a hurdle technology framework, complementing other preservation strategies to ensure microbial safety and respond to consumer demand for minimally processed, natural, and safe meat products (Da Costa et al., 2019; Fernandes et al., 2024).

#### 3.1.1 Class I bacteriocins (lantibiotics)

This type of bacteriocin consists of one or two small peptides (<5 kDa). Further, it is a post-translational modified bacteriocin. Hence, it consists of unusual amino acids like lanthionine, β-methyllanthionine, and dehydrated amino acids, which facilitate the structural stability for heat, pH, and proteolytic resistance. Furthermore, it commonly inhibits foodborne pathogens and gram-negative bacteria. Moreover, class I bacteriocins are again classified as Group Ia and Group Ib. Here, group Ia bacteriocins (nisin, epidermin, gallidermin) are positively charged peptides that form pores in the bacterial cell wall, thereby increasing the permeability in target bacteria, thus destroying the pathogens. Group Ia bacteriocins are screw-shaped, flexible, and amphipathic. Group Ib bacteriocins are negatively charged peptides that inhibit enzyme activity and kill the target bacteria. Examples of these bacteriocins are lacticin 481, cytolysin, and salivaricin, and they are globular-shaped and inflexible (Kaveh et al., 2023; Perez and Ancuelo, 2023; Lahiri et al., 2022a).

#### 3.1.2 Class II bacteriocins (small non-lantibiotics)

Class II bacteriocins are small, stable peptides that are hydrophobic and resistant to heat. They typically consist of 30–60 amino acids and have a molecular weight of less than 10 kDa. These peptides exhibit an amphiphilic helical structure, which plays a crucial role in disrupting bacterial membranes, resulting in depolarization and ultimately leading to pathogen cell death. Since they lack lanthionine or methyllanthionine, they are classified as non-lantibiotics (Alvarez-Sieiro et al., 2016). The unusual amino acids that are present in class I bacteriocins are not present in class II bacteriocins. The post-translational modification causes bisulfide bridge formation in some bacteriocins, for example, pediocin PA-1 and pediocin AcH. Like class I bacteriocins, class II bacteriocins are also heat-stable, <10 kDa-sized peptides that cause larger pores on the bacterial surface, thus increasing their ease of entry into the bacterial cells, destroying them. Further, it was divided into four subclasses. Group IIa bacteriocins are linearly structured and consist of bisulfide bridges, which can inhibit or kill *Listeria* sp. Hence, it is called an anti-listerial bacteriocin, which includes leucocin A, acidocin A, and pediocin PA-1. Group IIb bacteriocins are called two-peptide bacteriocins, which have antimicrobial activity, and include lactococcin G, lactococcin Q, and plantaricin NC8. Group IIc bacteriocins are leader peptide sequences containing one or two cysteine residues, cystibiotics and thiolbiotics, respectively, which are known for their antimicrobial activity. Group IId bacteriocins are linear, non-pediocin-like, single-peptide bacteriocins which include epidermicin NI01 and lactococcin A (Yang et al., 2014; Abdul Hakim et al., 2023).

#### 3.1.3 Class III bacteriocins (large non-lantibiotics)

Class III bacteriocins, which exceed 30 kDa in size, are produced by *Lactobacillus helveticus* and are heat-labile. These bacteriocins, like those synthesized by other bacteria, must be secreted to engage with target cells and exert their antimicrobial properties (Wang et al., 2021). Furthermore, this group was divided into two subgroups, designated as group IIIa and IIIb. Group IIIa is a lytic bacteriocin like Lysostaphin, which can lyse the cell membrane and thus destroy the bacterial cell. Moreover, the group IIIb bacteriocins, like Enterolysin A, disrupt the cell wall and reduce intercellular ATP concentration, resulting in the death of bacteria (Raman et al., 2022; Xu J. et al., 2025).

#### 3.1.4 Class IV bacteriocins (complex conjugates)

A fourth group was traditionally proposed for complex bacteriocins with lipid or carbohydrate moieties (lipoprotein or glycosylated conjugates). However, these are controversial, as many lack a clear demonstration of ribosomal peptide origin and consistent antimicrobial activity. Modern consensus generally excludes Class IV from the bacteriocin framework, treating Classes I to III as the accepted groups. Class IV is best regarded as a deprecated or provisional category (Alvarez-Sieiro et al., 2016; Lahiri et al., 2022b; Solis-Balandra and Sanchez-Salas, 2024).

### 3.2 Enzymes

Enzymes are biocatalysts that are significantly involved in all anabolic and catabolic pathways, and LAB effectively produce some of these enzymes, including lactase, proteases, peptidases, fructanases, bile salt hydrolase, and phytases. Lactase, also called β-galactosidase enzyme, used in the milk industry, can degrade lactose molecules into glucose and galactose. Lack of this enzyme in human beings causes a health issue called lactose intolerance. The lactase produced by LAB is considered an excellent solution for lactose indigestion. Various species like *Lactobacillus delbrueckii* subsp. *bulgaricus* and *Streptococcus thermophilus* can produce the highest concentration of lactase (Ayivi and Ibrahim, 2022). The proteolytic enzymes produced by LAB include proteinases, peptidases, and transport proteins. Proteinases are involved in the degradation of casein in milk products into peptides. Further, peptidases cleave the peptides into amino acids and smaller peptides. The transport protein transfers amino acids and peptides across the cytoplasmic membrane (Kieliszek et al., 2021). It has been reported that some LABs produce fructanases, which break down fructan into fructose and add sugar to bread (Murniece et al., 2025). Similarly, Kusada et al. (2022) reported that bile salt hydrolase produced by LAB can hydrolyse glycine/taurine-conjugated bile salts produced by mammalian digestive tracts. The LAB-produced phytase breaks down phytate and releases myo-inositol, lesser forms of inositol phosphate, and solubilized forms of inorganic phosphate (Sharma et al., 2020). In addition to proteins, lipids, and glycogen in meat products, LAB degraded dietary compounds (Wang et al., 2021). Thus, LAB are involved in the production of various enzymes and play a crucial role in the digestion of various food products.

Enzymes play both endogenous and exogenous roles in meat processing. After slaughter, endogenous proteases such as calpains, cathepsins, and their regulators (e.g., calpastatin) gradually break down muscle proteins during aging or maturation, improving tenderness, juiciness, and flavor of the meat (Abril et al., 2023). To accelerate or control these changes, exogenous enzymes, particularly proteases from plant sources like papain, bromelain, ficin, actinidin, zingibain, and others, are applied to tougher or lower-quality meat cuts (Mohd Azmi et al., 2023; Abril et al., 2023; Fayaz et al., 2024). These proteolytic enzymes cleave structural proteins in myofibrils and connective tissue, reducing toughness and improving palatability (Mohd Azmi et al., 2023; Fayaz et al., 2024). Enzymes like transglutaminase are used to bind small meat pieces together, reducing waste and creating restructured products with better texture. Additionally, enzymatic control of glycolysis, lipolysis, and proteolysis during processing influences flavor development, color stability, and overall quality attributes. However, practical challenges such as enzyme stability, control of over-digestion (which can cause mushy texture), cost, and compatibility with other processing steps must be managed carefully (Mohd Azmi et al., 2023; Abril et al., 2023; Fayaz et al., 2024).

### 3.3 Gamma-aminobutyric acid

Gamma-aminobutyric acid (GABA), a neuroinhibitory amino acid, is naturally found in plants and mammals. GABA’s natural abundance in plants, foods, and mammalian tissues is generally low, necessitating chemical production or microorganism-based bioconversion (Wu and Li, 2018). LAB produce GABA via the glutamate decarboxylase (GAD) pathway, in which the enzyme GAD catalyzes the decarboxylation of L-glutamate (or its salt, e.g., monosodium glutamate) to yield GABA and CO₂. Many GABA-producing LAB strains carry one or more gad genes (e.g., *gadA, gadB*) and often a glutamate/GABA antiporter (*gadC*) to export GABA out of the cell (Yogeswara et al., 2020; Lyu et al., 2018; Diez-Gutiérrez et al., 2022; Cataldo et al., 2024). The process of converting glutamate to GABA is not irreversible. There is data in the literature demonstrating the use of GABA as an energy source by microorganisms. GABA can enter the tricarboxylic acid cycle and be converted to glucose. Furthermore, its production by LAB does not occur solely through the action of GAD. It can also be produced through the metabolism of putrescines (Cui et al., 2020; Diez-Gutiérrez et al., 2020).

The production of GABA by LAB is often associated with acid stress/acid tolerance mechanisms. Under low pH or acid challenge (such as during fermentation or in acidic environments), activation of the GAD system helps the cell consume intracellular protons (H^+^) through the decarboxylation reaction, thus contributing to maintaining intracellular pH homeostasis (Dhakal et al., 2012; Cui et al., 2020; Diez-Gutiérrez et al., 2022; Cataldo et al., 2024). In many LAB (e.g., *Lactobacillus brevis*), expression of the gad operon is upregulated at lower pH, linking GABA production to survival under acidic conditions (Lyu et al., 2018; Cataldo et al., 2024).

GABA is considered one of the bioactive compounds produced by LAB, which may be beneficial to the user’s health. By enhancing oxygen delivery and blood flow, GABA can improve the metabolism of brain cells that regulate blood pressure, protein synthesis, hormone production, and fat burning (Alizadeh Behbahani et al., 2020). Since the food industry prohibits the use of chemically manufactured GABA, bioconversion employing food-grade LAB has emerged as a crucial technique for producing GABA or GABA-rich foods (sprouted or germinated grains and legumes, and fermented foods like kimchi, yoghurt and cheese, especially when fermented with GABA-producing LAB) (Lee and Paik, 2017). LAB produces GABA by utilizing the enzyme glutamate decarboxylase to decarboxylate L-glutamate in an anaerobic environment, while also utilizing protons (Woraharn et al., 2014; Tang et al., 2023). Potential health benefits of GABA include lowering cholesterol, regulating blood pressure, having anti-carcinogenic qualities, and preventing depression by encouraging relaxation and lowering anxiety. *Lactobacillus namurensis* (Reclassified as *Levilactobacillus namurensis*), *Lactobacillus paracasei* (Reclassified as *Lacticaseibacillus paracasei*) and *Lactobacillus brevis* (Reclassified as *Levilactobacillus brevis*) are examples of LAB species that have demonstrated the ability to produce GABA through glutamate decarboxylase (Alizadeh Behbahani et al., 2020).

### 3.4 Short-chain fatty acid

The human intestine lacks some of the carbohydrate digestive enzymes, which affects gut health. Generally, cellulose, xylans, resistant starch, inulin, and dietary fibers often remain undigested by the human intestine due to a lack of digestive enzymes. These compounds are denoted as undigested carbohydrates. LAB can ferment this kind of undigested carbohydrate into short-chain fatty acids (SCFAs), including butyric acid, propionic acid, and acetic acid. These SCFAs have therapeutic applications and play a crucial role in maintaining gut health. Acetic acid is essential for controlling intestinal inflammation and plays a role in minimizing the spread of pathogens. Similarly, butyric and propionic acid can improve insulin responsiveness and decrease the risk of diet-induced obesity. Furthermore, butyric acid also exhibits anticancer activity against colon cancer (Pessione et al., 2015). Moreover, the formation of SCFAs in the gastrointestinal region creates an acidic environment, resulting in the depletion of growth of harmful bacteria and plays a vital role in diminishing the proliferation rate of harmful bacteria (LeBlanc et al., 2017). It has been reported that *Lactobacillus plantarum* (Reclassified as *Lactiplantibacillus plantarum*)*, Lactobacillus pentosus* (Reclassified as *Lactiplantibacillus pentosus*), and *Leuconostoc mesenteroides* are the strains capable of effectively producing SCFAs with notable antibacterial properties (Pessione et al., 2015). Additionally, *Lactiplantibacillus plantarum* has been shown to degrade lipid compounds in meat, leading to the formation of SCFAs (Uppada et al., 2017).

### 3.5 Organic acids

LAB are known for their important role in fermentation, producing a variety of organic acids that are significant metabolic products (Von Wright and Axelsson, 2019; Chen et al., 2017). Depending on the metabolic pathway, some metabolisms, such as sugar metabolism, can produce different types of organic acids, including lactic acid, acetic acid, butyric acid, and propionic acid. The primary result of the metabolic pathway is lactic acid, which is then separated into l-lactic acid and d-lactic acid according to the various arrangements of the chiral atom. Lactic acid is produced due to anaerobic conditions throughout the glycolysis pathway, contributing to the sour taste of fermented foods (Chen et al., 2017). These organic acids influence the taste, consistency, and shelf life of fermented foods while also promoting food safety by preventing the growth of pathogens and spoilage organisms. The primary anti-bacterial actions of these acids result from their disruption of the bacterial cytoplasmic membrane, which impairs active transport pathways and disrupts the membrane potential, ultimately inhibiting the growth of harmful microorganisms (Bangar et al., 2022). Lactic acid is essential to the fermentation of foods, including cheese, yogurt, pickles, and sauerkraut (Ayivi and Ibrahim, 2022). LAB produces it, specifically *Lactobacillus* (Reclassified as *Lacticaseibacillus, Levilactobacillus, Ligilactobacillus*, and *Lactiplantibacillus*) and *Streptococcus* species, which break down carbohydrates, including lactose in milk, through metabolic processes (Bangar et al., 2022).

### 3.6 Vitamins

Vitamins are micronutrients that are involved in human metabolism, but humans are unable to synthesize them. Hence, food materials are considered the sole source of vitamins in humans. Many LABs can produce various vitamins, such as vitamin B and vitamin C. During lactic acid fermentation, vitamins are produced by LABs, which play a vital role in the production of nutrient-fortified food products. Folic acid or vitamin B9 is significant for the biosynthesis of nucleotides, DNA, RNA, and proteins. It has been demonstrated that *Lactococcus lactis* and *Streptococcus thermophilus* are capable of synthesizing folic acid in the human gut, which serves as a precursor for nucleotide and nucleic acid biosynthesis (Sybesma et al., 2003). Many LABs possess riboflavin (vitamin B2) synthase genes such as ribG, ribB, ribA, and ribH within their operon, which catalyze the production of riboflavin using guanosine triphosphate and ribulose-5-phosphate as substrates. Furthermore, it has been noted that *Lactiplantibacillus plantarum* CRL 725 can produce riboflavin (Juarez del Valle et al., 2014). Additionally, Li et al. (2017) indicated that vitamin B12 can be sourced from meat and meat-derived products (Bacon, sausage, ham, and other animal-source foods like milk and eggs).

Vitamin C is a water-soluble vitamin with high antioxidant potential, playing a crucial role in maintaining human health. During lactic acid fermentation, the LAB can produce Vitamin C (Quan et al., 2022). LABs like *Lactiplantibacillus plantarum, Limosilactobacillus fermentum, Lactobacillus acidophilus,* and *Bifidobacterium longum* can produce vitamin C. Moreover, LAB can also synthesize vitamin K2 (menaquinones) via the menaquinone-synthesis pathway. *Lactococcus lactis* has the potential to produce vitamin K2 using various carbon sources (fructose, trehalose, maltose, and mannitol). Considering the crucial role of LAB in the production of vitamins, it has also been used in recent years for therapeutic applications to reduce vitamin deficiency or inflammatory diseases (Liu et al., 2019).

### 3.7 Exopolysaccharides

Exopolysaccharides (EPS) are polysaccharides produced by microbes. They are expelled from the bacterial cell wall. LABs are the ones that create the most different types of EPS (Sanalibaba and Cakmak, 2016). For fermented foods to have their specific texture, viscosity, and probiotic qualities, EPS is essential. Due to their ability to retain water, these polymers are commonly used in the food industry as stabilizers and emulsifying agents (Singh and Saini, 2017). Conversely, EPS have been linked to the potential health advantages of their anti-inflammatory, antitumor, and anticancer properties. Numerous studies have demonstrated that EPS support gut health and encourage bacterial colonization by creating a protective matrix (Flemming, 2016). *Weissella, Leuconostoc, Lactococcus, Fructilactobacillus,* and *Lactiplantibacillus plantarum* are particularly capable of producing various types of EPS, depending on the strain. Environmental elements that affect EPS production include pH, temperature, time, and the LAB strain (Angelin and Kavitha, 2020).

Depending on the makeup of the sugar unit, these polymers can be divided into homopolysaccharides (HoPS) and heteropolysaccharides (HePS). HePS are made up of various kinds of monosaccharides, while HoPS are polysaccharides made up of a single type of monosaccharide. The species of lactic acid bacteria that contribute to the broad range of uses in the food industry determine the sugar composition and chain length of the EPS (Korcz and Varga, 2021). Numerous enzymes and regulatory proteins are involved in the intricate process of bacterial EPS biosynthesis. The biosynthesis of EPS can be broadly divided into three stages: First, the carbon substrate is taken up. Subsequently, the polysaccharides undergo intracellular synthesis before being excreted from the cell. Sugar transfer into the cytoplasm, sugar-1P synthesis, polymerization of repetitive unit precursors, and EPS transport outside the cell are the first four major processes in the biosynthesis of EPS in LAB (Becker, 2015). Among the key features of HoPS synthesis are the absence of active transportation phases in the synthetic process, the requirement for extracellular enzyme production, and the minimal energy expenditure. These extracellular enzymes are known as fructosyltransferases and glycosyltransferases. Glycansucrases are another name for glycosyltransferases. Glucose is used by this enzyme. Another name for fructosyltransferase is fructansucrase. Moreover, this enzyme uses fructose. When HoPS is being synthesized, these sugars serve as the glycosyl donor (Juvonen et al., 2015).

Furthermore, the manufacture and secretion of HePS include several proteins and/or enzymes. The production of HePS depends on the sugar nucleotides. The two main functions of these sugar nucleotides, which are produced from sugar-1-phosphates, are (1) sugar activation (monosaccharide polymerization requires sugar activation), and (2) sugar interconversions (epimerization, decarboxylation, dehydrogenation, and so on). The biosynthesis of HePS is an energy-intensive process. This process involves several energy-consuming steps: (1) the conversion of sugar-to-sugar phosphate requires one ATP, (2) each nucleotide requires another, and (3) the phosphorylation of the isoprenoid C55 lipid carrier requires an additional ATP (Madhuri and Prabhakar, 2014).

### 3.8 Bioactive peptides

The proteases and peptidases produced by humans can release bioactive peptides from encrypted proteins, which are then absorbed by the human gut and other peripheral organs. The enzymatic activity of LAB significantly influences the release of peptides from proteins and thus increases the digestion in humans. However, LAB possesses a limited genome length, and they have restricted capabilities in synthesizing amino acids. Hence, LAB adopted a complex and sophisticated proteolytic system to convert the external protein into amino acids and small peptides. Generally, bioactive peptides have the following beneficial effects in humans, including antimicrobials, hypocholesterolemia, opioid antagonists, angiotensin-converting enzyme inhibitors, anti-thrombotic, immunomodulators, cytomodulators, and antioxidants (Perez and Ancuelo, 2023; Mazorra-Manzano et al., 2020). As reported by Fan et al. (2019), *Lactobacillus helveticus* CICC6024 produces nearly 241 bioactive peptides under defined fermentation conditions. This corroborates the recent findings of Carneiro et al. (2024), indicating that LAB can synthesize bioactive peptides from meat and meat products.

## 4 LABs for the meat product preservation and safety

Human health and the economy were greatly affected by foodborne infections and intoxications. For the past several decades, various chemical preservatives have been employed in the food industry, which cause various toxic effects and diseases, including allergic reactions, heart disease, neurological problems, and cancer. Hence, to replace the chemical preservatives, biopreservatives like microorganism and their metabolites were used to make safe food for consumers. Further, worldwide consumers prefer products that do not contain chemical preservatives. Biopreservatives enhance the safety, quality, and shelf life of food items by inhibiting the growth of harmful microorganisms through the antagonistic activity of LAB, which is witnessed through the production of organic acids, hydrogen peroxide, diacetyl, bacteriocins, and other low-molecular-weight metabolites (Sharma et al., 2022).

Generally, LAB can eliminate various food spoilage-causing bacteria, such as *Escherichia coli, Salmonella* sp., and *Listeria monocytogenes*, which generally grow on the surface of meat products, thus spoiling their quality (Castellano and Vignolo, 2006). Specific spoilage organisms, such as *Pseudomonas* sp. *Brochothrix thermosphacta, Enterobacteriaceae* spp., *Acinetobacter* spp., *Aeromonas* spp., *Alcaligenes* spp., *Moraxella* spp., *Flavobacterium* spp., *Staphylococcus* spp., and *Micrococcus* spp. were found to grow predominantly on the meat surface. Thus, spoiling the quality of meat, including its color, texture, appearance, and flavor, makes the meat product undesirable or unfit for human consumption (Marcelli et al., 2024). However, the usage of LAB can promptly reduce the load of food spoilage organisms, thus enhancing its shelf life (Sharma et al., 2022).

It was evident from the previous study that LABs (*Lactiplantibacillus plantarum, Levilactobacillus brevis,* and *Leuconostoc mesenteroides*) isolated from poultry meat can produce lactic acid, hydrogen peroxide, and diacetyl, thereby inhibiting the growth of various pathogenic organisms (Adesokan et al., 2008). *Lactiplantibacillus plantarum* and *Leuconostoc mesenteroides* have antagonistic activity against *Staphylococcus aureus, Pseudomonas aeruginosa,* and *Escherichia coli*, thus helping to prevent meat spoilage. LAB produces lactic acid as a primary metabolite, which reduces the pH of the food product. Hence, in the acidic environment, the growth of foodborne microorganisms is inhibited by affecting their cell membrane and thus making the food product fit for human consumption (Lahiri et al., 2022a,b; García-Díez and Saraiva, 2021).

LAB produces hydrogen peroxide (H_2_O_2_) through the action of the enzyme flavoprotein oxidase in the presence of oxygen. Since LAB lack the catalase enzyme, H_2_O_2_ can accumulate in the environment, which can oxidize the lipid membranes and cellular proteins of pathogenic organisms, such as bacteria, yeasts, molds, and viruses (Sharma et al., 2022).

Diacetyl (2,3-butanodione) is a volatile organic compound produced by LAB through citrate fermentation and can inhibit the foodborne pathogenic organisms. Diacetyl produced by LAB can prevent the growth of gram-negative bacteria, yeasts, and molds than gram-positive bacteria by deactivating the key enzymes in the pathogenic microbes (Silva et al., 2023).

*Lactococcus lactis* subsp. *lactis* I23 and *Lactococcus lactis* subsp. *lactis* E91 resides in its ability to produce lactic acid and diacetyl and inhibit *Brochothrix thermosphacta*, a meat spoilage organism in fresh pork (Olaoye et al., 2015). *Latilactobacillus curvatus* CRL705 and its bacteriocin compounds, such as lactocin 705 and lactocin AL 705, when introduced into fresh meat, were found to inhibit the growth of *Listeria innocua* and *Brochothrix thermosphacta in* vacuum-packaged fresh meat at 2 °C (Castellano and Vignolo, 2006).

Meat and meat products are rich in protein, vital amino acids, minerals, and vitamin B groups, making them excellent sources of nutrients for people. Additionally, due to their optimal pH, nutritional elements, and high-water activity, they provide a suitable environment for the growth of a diverse range of microorganisms (Bohrer, 2017). The genera *Brochothrix, Enterobacter, Acinetobacter, Moraxella, Pseudomonas, Leuconostoc,* and *Proteus* are primary causes of meat deterioration; however, some of these bacteria, such as *Enterobacter* and *Pseudomonas*, also release biogenic amines (BAs) that may compromise food safety (Gao et al., 2022). Biogenic amines (BAs) are nitrogen-containing compounds that are mainly generated through the decarboxylation of amino acids. While BAs play a crucial role in various biological functions, elevated levels can pose risks to human health. Significant amounts of BAs are commonly present in fish sauces and fermented sausages. Various chromatography techniques and chemosensors are employed to identify BAs in food products. Preventive strategies include the application of starter cultures, control of physical and environmental conditions, and the incorporation of polyphenols. To ensure food safety, it is essential to conduct regular monitoring, adhere to hygienic production methods, and utilize effective starter cultures (Sivamaruthi et al., 2021). Additionally, harmful microbes such as *Campylobacter jejuni, Salmonella* spp., *Yersinia enterocolitica, Bacillus cereus, Clostridium perfringens, Clostridium botulinum, Escherichia coli,* and *Listeria monocytogenes* can contaminate meat and animal products (Favaro and Todorov, 2017). One of the primary issues facing the meat industry is the spoilage of fresh meat and meat products due to microbial contamination (Ashaolu et al., 2023). The meat industry employs several techniques to prevent microbiological growth and produce safe products with the desired quality and intended storage time. As a result, the most used methods include chemical approaches (such as the use of artificial preservatives) and physical methods (such as drying, freezing, heat treatment, packaging, and curing). However, chemical additives have several drawbacks, including altering the nutritional and organoleptic properties of food (Kaveh et al., 2022; Radi et al., 2023).

In this regard, LAB have garnered greater interest than other bio-preservative microorganisms for a variety of reasons, including their ability to encapsulate via extrusion during the creation of the antimicrobial film and their generally recognized as safe classification, which allows the FDA to approve them as a preservative in certain foods (Radosavljević et al., 2022). Therefore, LAB is essential to the development of fermented meat products, which increase texture and flavor while also preserving the product and, ultimately, extending its shelf life. Fresh meat’s high buffering capacity and low carbohydrate content result in mild fermentation, without altering the organoleptic qualities of the food. LAB produces a variety of bioactive substances, including biosurfactants and bacteriocins, which are utilized to preserve meat products. Bacteriocins may inhibit the growth of spoilage or pathogenic microorganisms. LAB-derived bacteriocins have demonstrated strong antimicrobial effects across a range of meat products, significantly enhancing preservation and safety. In ready-to-eat pork ham, bacteriocin-like inhibitory substances from *Pediococcus pentosaceus* inhibit *Listeria seeligeri* by 1.74 log CFU/g and reduce weight loss (de Azevedo et al., 2020). Similarly, *Bacillus sonorensis*-derived sonorensin effectively inhibited *Listeria monocytogenes* and *Staphylococcus aureus* in inoculated chicken meat (Chopra et al., 2015). Vacuum-packaged beef frankfurters treated with semi-purified bacteriocins from *Latilactobacillus curvatus* or *Latilactobacillus sakei* exhibited pathogen levels reduced to below the detectable limit (Castellano et al., 2018). In beef, bacteriocins from *Lactobacillus crustorum* MN047 (Reclassified as *Companilactobacillus crustorum*) significantly reduced the populations of *Escherichia coli* and *Staphylococcus aureus* by 4.3 and 4.5 log CFU/ml, respectively (Lu et al., 2020). Antimicrobial peptides, especially bacteriocins generated by probiotics, offer a promising therapeutic strategy for combating infectious diseases. LAB strains with probiotic potential were isolated from fermented foods and assessed for their ability to produce EPS, their susceptibility to antibiotics, tolerance to acid and bile, antibacterial properties, and their adhesion/cytotoxicity to gastric cell lines. Six LAB strains were chosen based on their high survival rates in the gastrointestinal tract, significant EPS production, low cytotoxicity, and strong adhesion to gastric cells. Notably, *Weissella confusa* CYLB30, *Lactiplantibacillus plantarum* CYLB47, and *Limosilactobacillus fermentum* CYLB55 demonstrated strong anti-bacterial effects against multidrug-resistant strains of *Escherichia coli, Klebsiella pneumoniae, Pseudomonas aeruginosa, Salmonella enterica* serovar *choleraesuis, Enterococcus faecium,* and *Staphylococcus aureus* (Thuy et al., 2024). These findings collectively demonstrate that bacteriocins from LAB offer potent, natural bio-preservatives that can significantly enhance the microbial safety and quality of various meat products.

**Figure 1:**
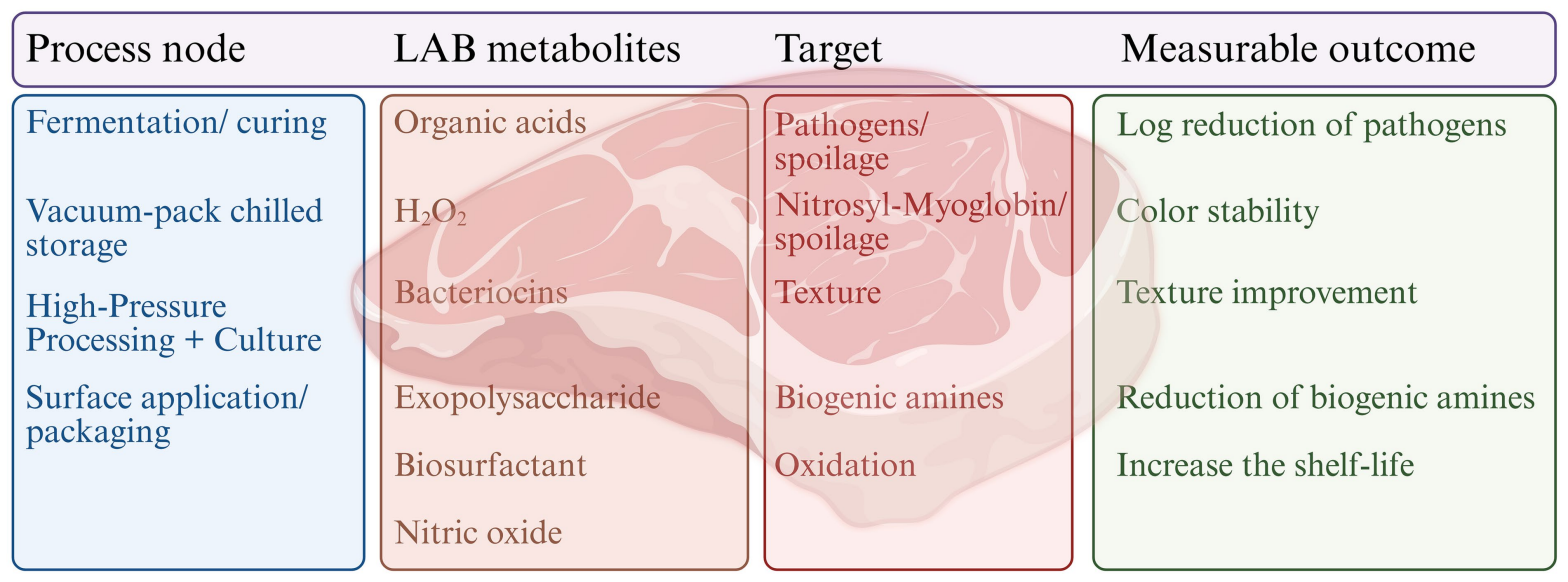
LAB metabolites with meat preservation processes and outcomes. Different process nodes influence the LAB metabolites, such as organic acids, hydrogen peroxide, bacteriocins, exopolysaccharides, biosurfactants, and nitric oxide. These metabolites target pathogens and spoilage organisms, nitrosyl-myoglobin stabilization, texture, biogenic amines, and oxidative reactions. Application of potent LAB could provide favorable outcomes, including pathogen reduction, color stability, texture improvement, decreased biogenic amine accumulation, and increased shelf-life of meat products.

**Figure 2:**
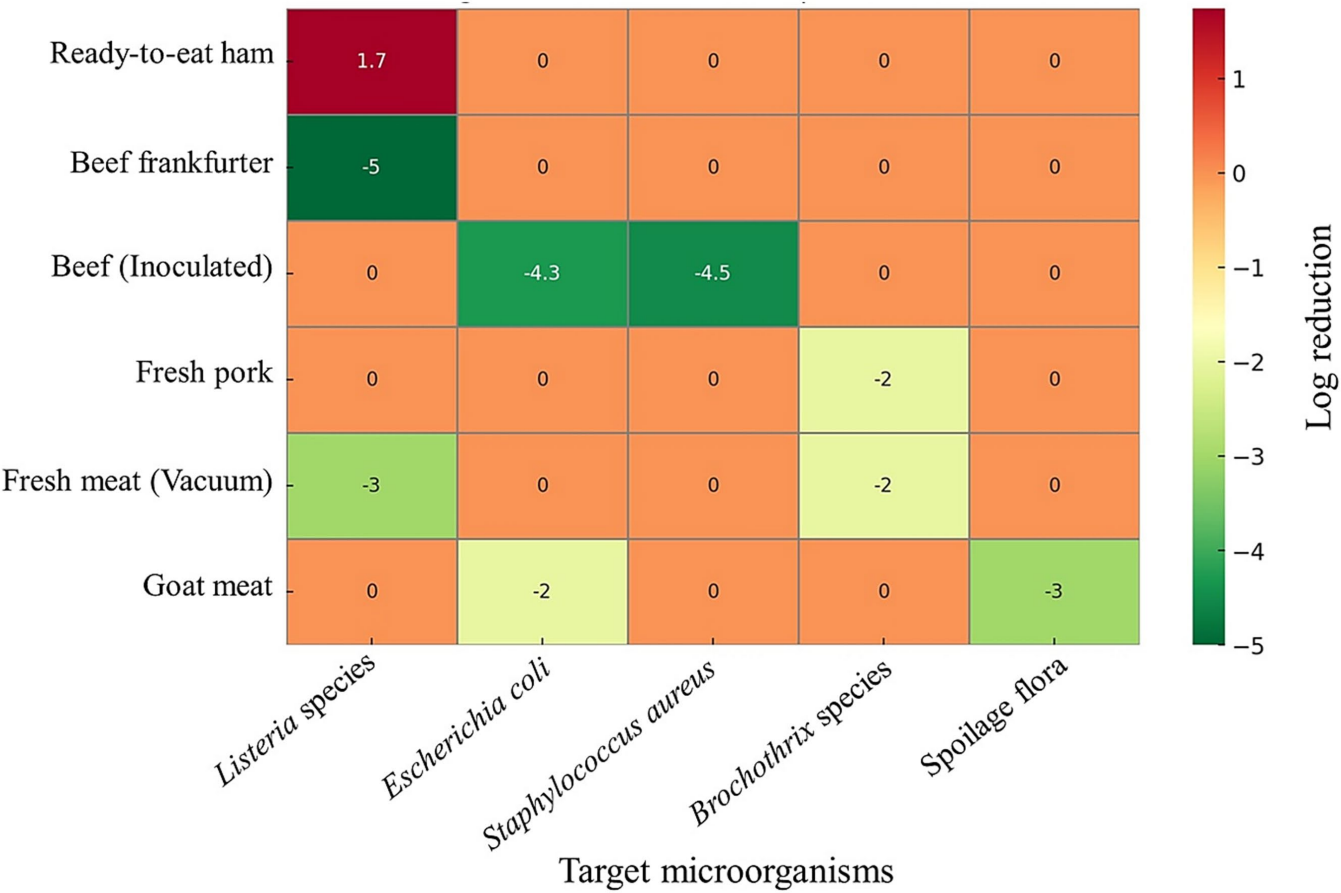
Log reduction of microorganisms in different meat matrices following LAB-associated interventions. The heatmap illustrates changes in microbial populations (log₁₀ CFU reduction or increase) across various meat products. Microorganisms tested were *Listeria* spp., *Escherichia coli*, *Staphylococcus aureus*, Brochothrix species, and general spoilage flora. Negative values (green) represent microbial reductions, positive values (red) indicate increases, and near-zero values (orange) denote negligible changes. The data has been entered in Microsoft Excel, and a heatmap was created using ChatGPT.

Bio-surfactants are amphiphilic, biodegradable, and non-toxic substances produced as secondary metabolites by various microbes, including lactic acid bacteria (Jahan et al., 2020). Food products, including meat products, can be effectively preserved due to their antibacterial properties. Through a variety of mechanisms, they demonstrate their antibacterial properties, including: (Abdul Hakim et al., 2023) the inhibition of bio-film formation by lowering the bacterial interaction with the surface by changing the surface’s charge and wettability (Ashraf et al., 2019); (Abril et al., 2023) interference with the microorganisms’ regular function by inter-action with their intracellular components (Ines and Dhouha, 2015); (Adesokan et al., 2008) destruction of the microorganisms’ cell walls and membranes (Hippolyte et al., 2018). Biosurfactants derived from *Lacticaseibacillus paracasei* and *Lacticaseibacillus casei* have demonstrated notable antimicrobial activity in meat preservation. In raw ground goat meat, these biosurfactants led to a significant reduction in total aerobic counts, including *Escherichia coli* MTCC 118 and *Pseudomonas aeruginosa* MTCC 1934 (Mouafo et al., 2020a, b). Similarly, in fresh beef, biosurfactants produced by *Lactobacillus paracasei* demonstrated complete inhibition of multiple spoilage and pathogenic bacteria, including *Bacillus* sp. BC1, *Staphylococcus aureus* STP1, *Staphylococcus xylosus* STP2 (Mouafo et al., 2020a, b). These findings underscore the potential of LAB-derived biosurfactants as effective natural antimicrobial agents for enhancing meat safety and extending shelf life. Figure 1 summarizes the linkage between LAB metabolites and practical effects in the meat industry. The heatmap (Figure 2) clearly shows how LAB metabolites reduce pathogens and spoilage organisms in different meats.

## 5 LAB as a quality enhancer of meat products

LABs have a significant advantage in the food fermentation and preservation process, enhancing the flavor, texture, aroma, digestible properties, and nutritional value of food products. The proteolytic and lipolytic effects of LAB convert protein and fat molecules into peptides, amino acids, and fatty acids, which enhance the flavor of food products. Hence, LAB paves a way for the development of preservation in the modern food biotechnology industries (Anumudu et al., 2024).

### 5.1 Flavor development

LAB strains, such as *Lacticaseibacillus, Limosilactobacillus, Leuconostoc,* and *Pediococcus*, can ferment various food compounds using their secreted enzymes to produce flavor precursors with complex sensory profiles. Carbohydrate fermentation, fatty acid metabolism, and amino acid catabolism are some of the significant metabolic processes carried out by LAB, which enhance the organoleptic properties of meat products by increasing tenderness and flavor (Anumudu et al., 2024). It has been reported that *Pediococcus acidilactici* BP2 enhanced the flavor of beef jerky (Wen et al., 2021). The raw meat contains skeletal muscles, which consist of myogenic fibrils, sarcoplasmic proteins, and matrix proteins. Further, LAB hydrolyses skeletal muscle proteins into oligopeptides. Subsequently, small peptides and amino acids are produced from oligopeptides and are further converted into α-keto acids and alcohols, which impart fruity flavors. Usually, aldehydes, alcohols, and aromatic substances are some of the flavor enhancement compounds that are produced via oxidative deamination and decarboxylation of proteins. Albano et al. (2009) stated that the flavor of a fermented meat sausage (Alheira) depends upon the LAB, quality of meat, and the ripening process.

The statistical analysis conducted by Xu B. et al. (2025) identified 47 volatile flavor compounds with sensory thresholds and 18 significant key flavor compounds with relative odor value activity values ranging from the relative odor activity (ROAV) value of 1 ≤ to ≤100 in sausage samples. These flavor compounds formed the distinctive flavor profile of Sichuan-style fermented sausages. The ROAV values for β-myrcene, caryophyllene, linalool, phenylethyl alcohol, 3-methyl-1-butanol, 1-octen-3-ol, 3-hydroxy-2-butanone, methyl isovalerate, methyl decanoate, 4-methoxy-6-(2-propenyl)-1,3-benzodioxole, anethole, and acetic acid were found to be higher in the five types of sausages that were inoculated with the combined starter cultures when compared to those in control group A. The contributions of β-myrcene, linalool, and anethole to the development of sausage flavor compounds were significant, suggesting that a greater number of flavor compounds were generated through microbial metabolism. Furthermore, the key flavor compounds such as acetic acid, caryophyllene, linalool, phenylethyl alcohol, 1,8-cineole, and 1-octen-3-ol in sausages inoculated with the combined starter culture F exhibited elevated ROAV values relative to the other compounds. It is hypothesized that *Debaryomyces hansenii* and *Latilactobacillus curvatus* present in the combined starter culture F facilitated the synthesis of key flavor compounds in Sichuan-style fermented sausages and enhanced the release of flavor compounds from spices.

### 5.2 Textural enhancement

LAB enhances the texture, sensory, and organoleptic qualities of meat products through various metabolic activities, including acidification, EPS production, and other enzymatic reactions. In meat and meat products, the protein and fatty acid compounds present in the muscle of meat undergo a gelation process due to the reduced pH caused by LAB, which enhances the disulfide bond formation in meat, thus increasing the chewiness of the meat (Anumudu et al., 2024). According to Smaoui et al. (2014), there was a reduction in hardness, springiness, and rigidity, increased adhesiveness, and chewiness in raw minced beef and chicken breast using BacTN635 (bacteriocin), extracted from *Lactiplantibacillus plantarum* sp. TN635. Similarly, Du et al. (2019) reported that LAB, such as *Pediococcus pentosaceus* and *Staphylococcus xylosus,* reduced the hardness of meat sausage.

### 5.3 Improvement of the color of the meat product

LABs are known to significantly enhance the coloration of meat products. For instance, *Lactiplantibacillus plantarum* has been demonstrated to reduce nitrite and nitrate to nitric oxide, which then reacts with myoglobin in sausages to form nitrosyl myoglobin, resulting in the characteristic pink color (Zhu et al., 2020). Similarly, *Lactobacillus fermentum* JCM1173 (Reclassified as *Limosilactobacillus fermentum*), *Limosilactobacillus fermentum* IFO3956, *Lactiplantibacillus plantarum* 8PA3, *Lactiplantibacillus plantarum* CMRC6, *Latilactobacillus sakei* CMRC15, and *Lactiplantibacillus plantarum* TN8 were identified as functionally active strains involved in the biochemical reduction of nitrate or nitrite to nitric oxide, thereby facilitating the formation of nitrosyl myoglobin and improving the color stability and appearance of meat products (Gou et al., 2019).

### 5.4 Enhancement of aroma

The amino acid and fatty acids were produced by proteolytic and lipolytic activity of LAB, which is further reduced to produce aroma-improving compounds such as alcohols, aldehydes, ketones, hydrocarbons, acids, aromatic compounds, esters, and sulfur-containing compounds. The free fatty acids are degraded into various compounds, including SCFAs (pungent and penetrating aroma) and secondary alcohols (fruity and fatty aroma). Whereas branched amino acids, including valine, isoleucine, and leucine, are decarboxylated to produce branched aldehydes, alcohols, and/or acids and cause malty and pungent aroma to meat. The aldehyde, alcohol, and acids from various amino acids, including phenylalanine, threonine, tryptophan, tyrosine, methionine, and cysteine, produce a fatty, tallow, malty, and fruity aroma to meat products (Flores, 2018).

## 6 Beneficial effects of lactic acid bacteria in meat products for consumer health

LABs are commonly used in food fermentation because they can preserve food. Nutritional and health advantages now influence consumer food preferences (Chaiyasut et al., 2018a), leading to decisions that increasingly favor the sustainable use of natural ingredients over chemicals as preservatives. This shift in consumer preferences has heightened the importance of utilizing LAB in food processing (Asioli et al., 2017).

**Figure 3:**
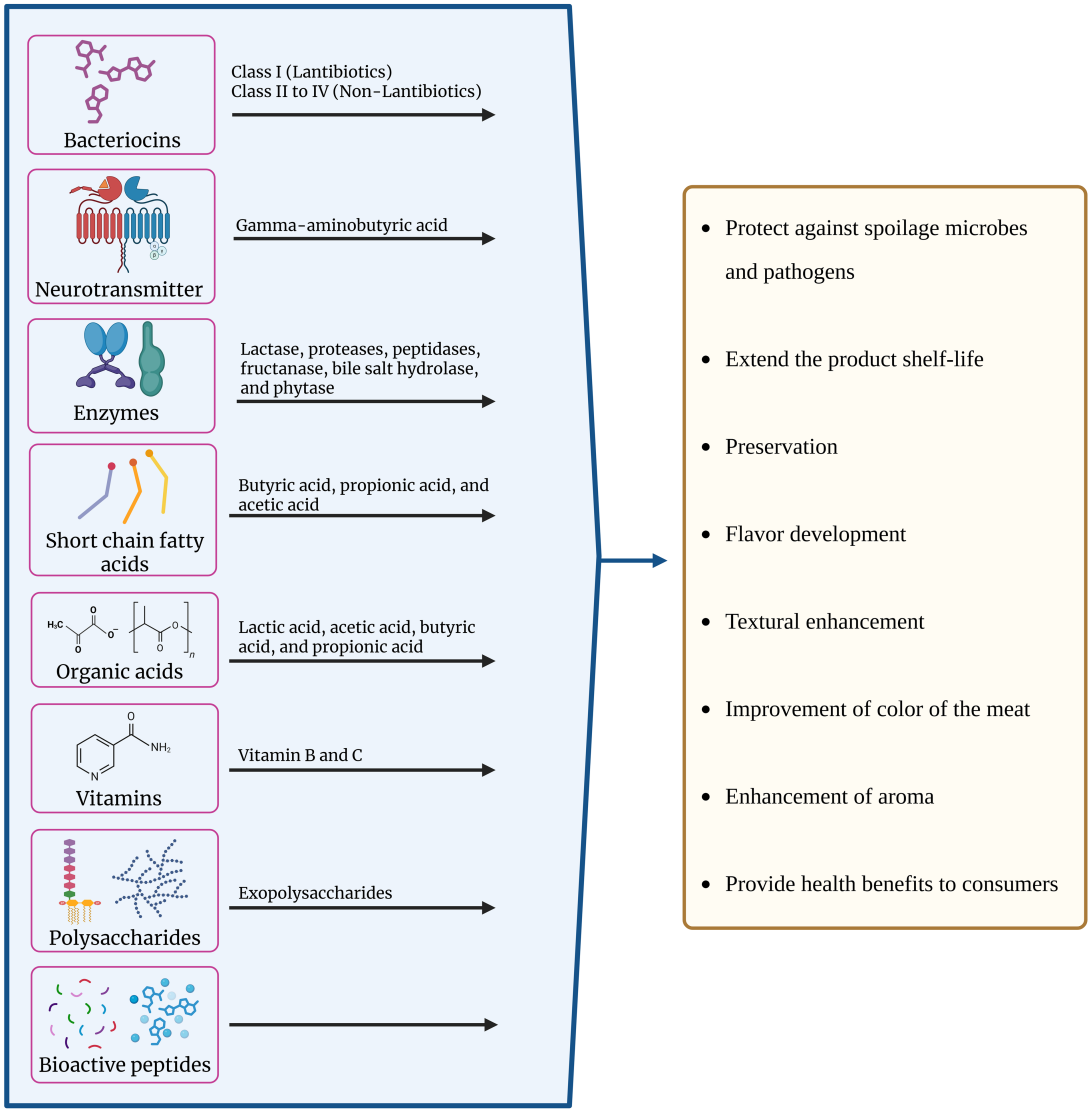
Bioactive metabolites produced by lactic acid bacteria (LAB) and their functional roles in meat products. LAB secrete a wide range of compounds including bacteriocins (Class I lantibiotics, Class II-IV non-lantibiotics), neurotransmitters (e.g., gamma-aminobutyric acid), enzymes (such as lactase, proteases, peptidases, fructanase, bile salt hydrolase, and phytase), short-chain fatty acids (butyric, propionic, and acetic acids), organic acids (lactic, acetic, butyric, and propionic acids), vitamins, polysaccharides (exopolysaccharides), and bioactive peptides. These metabolites collectively contribute to multiple technological and health-promoting effects in meat products, including protection against spoilage microbes and pathogens, extension of shelf-life, preservation, flavor and aroma development, textural enhancement, improvement of meat color, and provision of health benefits to consumers.

Meat fermentation is a complicated process from the perspective of its microbial ecology, where coagulase-negative staphylococci and LAB both play a role in the development of the product’s typical sensory qualities and its bio-preservation (Fraqueza, 2015). LAB can be included in the non-starter microbiota in fermented products or employed as probiotics and/or meat starter cultures, interacting with the product’s natural microbes. In both situations, their existence may benefit the results. Using starter cultures, which include probiotic bacteria with potential health benefits (Chaiyasut et al., 2018b; Chaiyasut et al., 2018c; Kesika et al., 2022; Sivamaruthi et al., 2022), supports consumer acceptance and the stability and safety of the product. Several factors should be considered when selecting LAB to produce fermented meats. Since they prevent the growth of pathogenic and deteriorating microorganisms, facilitate maturation, ensure microbial stability during storage, stabilize the product’s color, and improve its texture, they increase the safety and shelf life of the finished products. For this reason, the ability to acidify and grow at low pH values is desirable for potential starter cultures in the meat industry and for preventing spoilage. Proteolytic activity is another desired quality that is crucial to the development of flavor during fermentation, as in the case of raw sausage fermentation. LAB can have a beneficial effect on the breakdown of proteins during meat fermentation. The flavor of the sausage depends on the ability to further transform the resultant peptides into volatile molecules (Todorov et al., 2017). Another advantageous feature of LAB is its antibacterial activity, as it inhibits the growth of microorganisms that cause spoilage and foodborne pathogens, which are crucial for maintaining product safety, shelf life, and quality (Zhang et al., 2021). Other advantageous characteristics to consider when screening LAB for use in fermented meat products are their capacity to break down biogenic amines, especially in smoked meat products, which contain amines, cholesterol, and carcinogens, as well as their ability to regulate lipid oxidation (Shao et al., 2021). The active players from LAB and their significant role in the improvement of meat products and their impact on consumers’ health have been showcased in Figure 3.

## 7 Safety and regulatory frameworks

Even when a species is generally regarded as safe, individual strains may acquire antimicrobial resistance (AMR) genes, virulence factors, mobile genetic elements, or decarboxylase pathways that raise safety concerns in fermented meats. A defensible workflow, therefore, evaluates each production strain at the strain level using whole-genome sequencing (WGS), phenotypic assays, and regulatory frameworks (QPS/GRAS) [EFSA Panel on Additives and Products or Substances used in Animal Feed et al., 2018].

High-quality WGS can be used to confirm strain identity and genome integrity, including assessment of assembly quality, contamination, and taxonomic assignment. Raw sequence data were deposited, and reporting followed European Food Safety Authority (EFSA) requirements, which mandate disclosure of assembly metrics, accession numbers, and database versions for microorganisms intended for use in the food chain (European Food Safety Authority, 2024).

AMR can be assessed using curated databases (CARD with RGI, ResFinder, and PointFinder), with all hits reported alongside cut-off values and database versions. The absence of acquired AMR determinants could be considered essential for acceptability in line with EFSA guidance. For any AMR-like signals, the genetic context can be examined, including neighboring elements such as integrases, transposases, and origin of transfer sites, to determine whether they were chromosomal or plasmid-associated [Alcock et al., 2023; Alcock et al., 2020; Florensa et al., 2022; EFSA Panel on Additives and Products or Substances used in Animal Feed et al., 2018; EFSA Panel on Additives and Products or Substances used in Animal Feed, 2012].

Virulence factors in the strain can be screened against the Virulence Factor Database (VFDB), with all hits reported by identity and coverage and subsequently evaluated for biological plausibility within the genus (Liu et al., 2022). Mobile genetic elements can be examined by identifying plasmid replicons and regions using PlasmidFinder and PLSDB, with annotation of integrative conjugative elements and prophages. Particular attention was given to co-localization of antimicrobial resistance or virulence genes on mobile elements, which was considered high risk. In cases of uncertainty, filter-mating assays were performed to verify the absence of horizontal transfer under food-relevant conditions (Carattoli et al., 2014).

The potential for biogenic amine (BA) formation can be assessed both genomically and phenotypically. Genomic screening targeted decarboxylase gene clusters, along with associated transporters and regulators. Phenotypically, strains would be tested to confirm the absence of BA production in meat matrices or defined media (EFSA Panel on Biological Hazards, 2011).

Antimicrobial susceptibility of the strain can be evaluated by determining minimum inhibitory concentrations (MICs) using standardized methods and comparing the results against EFSA-established cut-off values. Concordance between genotypic predictions and phenotypic outcomes can be expected, and any phenotypic resistance exceeding cut-off thresholds required genetic justification or led to exclusion of the strain [EFSA Panel on Additives and Products or Substances used in Animal Feed et al., 2018; EFSA Panel on Biological Hazards, 2011].

Additional safety and fitness characteristics relevant to meat applications can be evaluated. For instance, acceptable profiles included γ-hemolysis only, with strains required to be negative for gelatinase, DNase, and genus-specific toxin activities. Spoilage potential was assessed through measurements of gas and H₂S production, detection of amine and aldehyde off-odors, and evaluation of proteolysis and lipolysis under target pH-salt-temperature conditions, with only non-spoiling strains retained. Furthermore, phage susceptibility mapping and prophage induction assays were performed to minimize risks of fermentation failure and horizontal gene transfer [EFSA Panel on Additives and Products or Substances used in Animal Feed et al., 2018].

Genetic stability and batch consistency need to be monitored by periodically re-sequencing the master cell bank and production seed lots to confirm the absence of new mobile elements, AMR determinants, or virulence factor genes. Traceability was ensured by maintaining versioned database records for all comparative analyses over time (European Food Safety Authority, 2024).

The proper strain informative documentation aligned with regulatory frameworks, noting that EFSA’s Qualified Presumption of Safety (QPS) operates at the species or group level, with specific qualifications (e.g., for production purposes only or absence of toxigenic activity) are needed. However, QPS designation does not exempt strains from detailed safety evaluation, including assessments of AMR, toxigenic potential, and suitability for the intended use. Therefore, the most recent QPS updates need to be consulted when selecting candidate species (EFSA Panel on Biological Hazards et al., 2024a; EFSA Panel on Biological Hazards et al., 2024b). In the U. S., GRAS status has been established for a specific microbial strain and its intended use in a food matrix, rather than assumed by species identity. A GRAS report typically includes strain characterization, safety assessments, history of use or toxicological evidence, and exposure estimates under intended conditions.

## 8 Research gap in the field

LABs play a crucial role in the meat industry, contributing to fermentation, preservation, and the enhancement of sensory attributes. They significantly contribute to food safety by inhibiting the growth of spoilage and pathogenic microorganisms through the production of antimicrobial compounds, including organic acids, bacteriocins, and hydrogen peroxide. However, despite their widespread use, several research gaps remain that require further exploration.

One major gap involves the strain-specific functionalities of LAB in meat products. While different LAB strains can influence texture, flavor, and preservation, their specific mechanisms and impacts are not fully understood. Identifying the best-performing strains for food applications would improve product quality and consistency. Another critical gap is related to the safety of LAB strains. Although many LAB species are considered safe for consumption, some may exhibit potential virulence properties, raising concerns about their long-term safety in food formulations.

For example, horizontal gene transfer (HGT) lets bacteria swap genes outside of parent-to-offspring inheritance and is a major driver of traits that threaten food safety. Foods can carry resistant bacteria and resistance genes that originated in animals or processing environments and later reach people; multiple studies have reported this pathway and its risks for human infection and risk of hard-to-treat infections (Lyu et al., 2018; Founou et al., 2016). Biofilms on food-contact surfaces are hotspots where bacteria easily swap plasmids, so hard-to-clean areas in processing plants and slaughterhouses can become long-term reservoirs of harmful genes (Van Meervenne et al., 2014; Ban-Cucerzan et al., 2025).

Concrete harms include the rapid spread of plasmid-mediated colistin resistance (mcr-1) from food animals into retail meat and human infections (Liu et al., 2016; Kuo et al., 2016). In food processing, *Listeria monocytogenes* frequently carries mobile determinants that raise tolerance to sanitisers such as benzalkonium chloride, aiding long-term facility persistence and recurrent product contamination (Dutta et al., 2013; Minarovičová et al., 2018; Daeschel et al., 2022). HGT by Shiga toxin-encoding phages can convert naive *Escherichia coli* into Shiga toxin-producing *Escherichia coli*, elevating virulence potential within the food chain and in the human gut (Khalil et al., 2016; Herold et al., 2004; Zuppi et al., 2020). Together, these routes show how HGT amplifies antimicrobial resistance and virulence across food systems, increasing outbreak risk and narrowing therapeutic options. Thus, further studies are needed to assess potential health risks and establish regulatory guidelines that ensure consumer protection.

Additionally, optimizing the production of beneficial metabolites, such as bacteriocins, organic acids, and bioactive peptides, remains a challenge. While these compounds contribute to antimicrobial activity and improved product stability, their yield and effectiveness vary depending on environmental conditions and bacterial strain. More research is needed to enhance their production efficiency and stability in industrial applications. Understanding the interaction between LAB and other microorganisms in meat products is another research area that remains underexplored. The presence of LAB can influence the growth dynamics of other bacterial populations, impacting the overall microbial balance and safety of meat products. Investigating these interactions would enable manufacturers to control undesired microbial activity and enhance food quality. Despite the documented benefits of LAB in meat products, consumer acceptance remains a challenge, especially in regions unfamiliar with LAB-enhanced meat. Public perception, taste preferences, and concerns about food safety significantly influence purchasing decisions, necessitating targeted studies on consumer attitudes and educational initiatives to enhance acceptance. Regulatory frameworks surrounding the use of LAB in meat products also require deeper investigation. While LABs are widely accepted in fermented products such as yogurt and cheese, their application in meat is still evolving, and clear guidelines for their use, labeling, and health claims need to be established.

Addressing these research gaps through interdisciplinary studies that involve microbiology, food science, biotechnology, and consumer behavior will enhance the safe and effective use of LAB in the meat industry.

## 9 Conclusion

LABs have become essential players in modern food biotechnology, especially in meat processing and preservation. The broad application of LABs stems from their ability to enhance food safety, extend shelf life, improve sensory qualities, and support human health. As natural fermenters, LAB contribute to the production of fermented meat products by generating organic acids, peptides, and bacteriocins that inhibit the growth of spoilage and harmful microorganisms, thereby reducing reliance on synthetic preservatives. This aligns with the growing consumer demand for “clean label” and minimally processed foods.

In meat products, LAB provides both technological and nutritional benefits. From a technological standpoint, they promote product stability through acidification, preservation, and enzymatic activity, which collectively enhance flavor, texture, and color. Nutritionally, certain LAB strains offer probiotic benefits, including modulation of the gut microbiota, cholesterol reduction, and support for the immune system. Additionally, they produce functional compounds, such as GABA and EPS, further enhancing the health-promoting potential of LAB-fermented meat products. Despite their advantages, LABs face challenges that require further research. Their strain-specific behavior in different meat matrices, interactions with native microbiota, and adaptation to processing conditions need deeper exploration. While LABs are GRAS, some strains may carry undesirable traits, such as antibiotic resistance or virulence factors, making rigorous safety assessments crucial for their industrial use. Another hurdle is scaling up the production of LAB-derived bioactive compounds without compromising their effectiveness in industrial applications. Consumer awareness and regulatory clarity also play a significant role. Acceptance of LAB-based innovations in meat products varies across cultures and markets, influenced by concerns over microbial safety and a lack of familiarity with fermented meats. Clear labeling, well-supported health claims, and targeted educational efforts are necessary to improve market penetration and consumer trust.

In summary, LAB presents a sustainable and effective approach to meat preservation and enhancement. Through continued interdisciplinary research that addresses safety, functionality, and consumer perception, LABs have the potential to transform the meat industry by meeting technological demands and public health needs in a natural and environmentally friendly manner.
